# Duplicated Descending Aorta Resulting in Early Localized Vascular Disease

**DOI:** 10.7759/cureus.38308

**Published:** 2023-04-29

**Authors:** John M Ricely, Shalom Mammen, Chandra Dass, Maruti Kumaran

**Affiliations:** 1 Diagnostic Radiology, Temple University Hospital, Philadelphia, USA; 2 Diagnostic Radiology, Temple University Lewis Katz School of Medicine, Philadelphia, USA

**Keywords:** ct angiography, diagnostic radiology, vascular disease, vascular anomaly, congenital aortic anomaly

## Abstract

While congenital variants of the aortic arch have been well described, anatomic anomalies of the descending aorta are extremely rare. We present a case of a 31-year-old male with congenital duplication of the descending aorta resulting in advanced localized atherosclerotic disease found incidentally on diagnostic imaging. This case presents a rare anatomic variant that can not only lead to early aortic disease but may also complicate future endovascular intervention.

## Introduction

Congenital abnormalities of the aortic arch are well documented; however, anomalies of the descending are rare, with only a few cases reported in the literature [1, 2]. The low incidence of congenital anomalies of the descending aorta may be attributed to the simplicity of its embryologic development, which is relatively less complex than that of the aortic arch. Although descending aortic anomalies may be asymptomatic, irregular anatomy can lead to increased wall stress and ultimately early vascular disease. Here, we present the case of a 31-year-old male with congenital duplication of the descending aorta, resulting in advanced localized atherosclerotic disease.

## Case presentation

**Figure 1:**
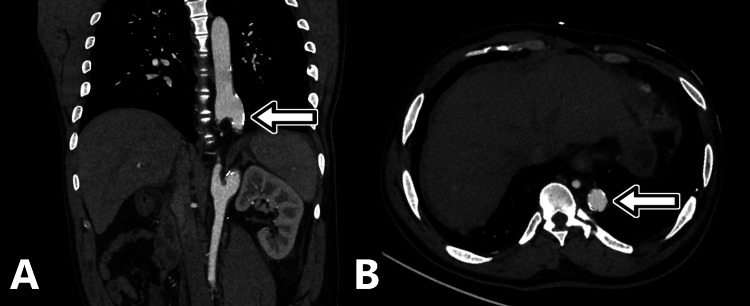
Coronal and axial CT angiography. The coronal projection (A) demonstrates both sites of bifurcation at the inferior descending aorta and superior abdominal aorta. The patent trunk (arrow) is ectatic with atherosclerotic calcifications.

**Figure 2:**
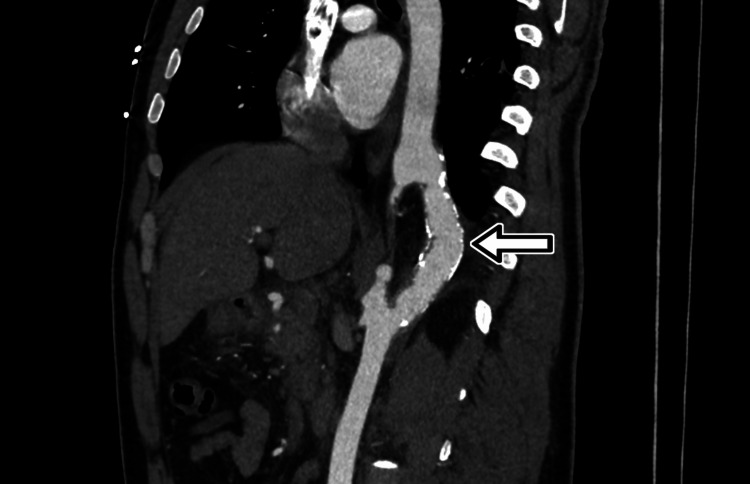
Sagittal CT angiography. The sagittal reformation demonstrates the full course of the patent trunk (arrow).

**Figure 3:**
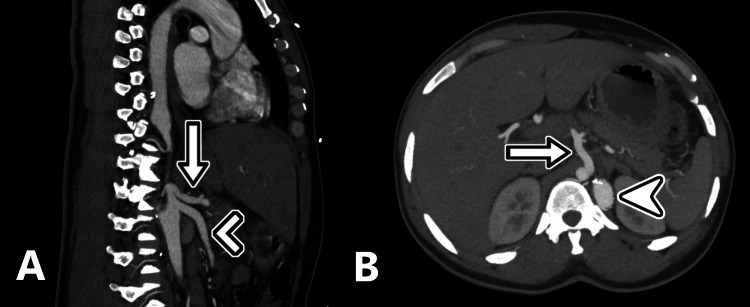
Sagittal and maximum intensity projection (MIP) axial reformation (arrowhead). The inferior portion of the rudimentary aortic trunk is shown giving rise to the celiac axis (arrow) and superior mesenteric artery (chevron). The patent trunk is also visualized on the MIP axial reformation (arrowhead).

A 31-year-old male with a past medical history of cerebral aneurysm repaired during childhood, alcohol abuse, and recurrent pancreatitis presented with three days of chest and abdominal pain. CT angiography of the chest, abdomen, and pelvis was performed due to concern for aortic dissection. The imaging was negative for aortic dissection but incidentally revealed an anomalous bifurcation in the descending aorta which led to a rudimentary right trunk and patent left trunk (Figure 1). The right trunk was atretic, while the left trunk remained patent but had a tortuous course and advanced localized atherosclerotic disease (Figure 2). The atretic trunk supplied the celiac axis and superior mesenteric artery (Figure 3) from retrograde flow via the patent trunk.

## Discussion

The aorta arises from the embryologic paired aortae, which comprise the primitive paired ventral aortae, aortic arches, and dorsal aortae. The ventral aortae form the aortic sac, the segment that communicates with the primitive heart tube. Caudally, the paired dorsal aortae fuse early in embryologic development to form the descending aorta [3].

While the aortic arch and great vessels form through a complex series of primitive arches which form early in embryogenesis and regress during fetal development, the development of the descending aorta is relatively simple. This explains why congenital anomalies of the descending aorta are so rare in comparison to arch anomalies. Given the embryologic development of the descending aorta discussed above, the anomaly seen in the patient presented here most likely represents an incomplete fusion of the primitive dorsal aortae rather than true duplication.

Turbulent flow is a known risk factor for the development of vascular disease [4], specifically atherosclerosis. Because the aorta is a low-resistance system, even modest variations in the course or contour can result in turbulent blood flow [5] and ultimately localized atherosclerosis, as seen in the patient presented here. Given the typical association of vascular disease with old age, identifying such an anomaly is critical to ensuring vascular pathology is included in the differential when symptoms develop. Further, the variant anatomy may pose a challenge to future endovascular interventions.

## Conclusions

Congenital anomalies of the descending aorta are exceptionally rare but are a potential cause of early vascular disease. The anatomy shown in this case reveals the embryologic basis for duplication of the descending aorta and highlights the early vasculopathic changes that may result from abnormal morphology. Such anomalies should be described in detail as they may influence the workup of certain symptoms or even preclude future endovascular interventions.
